# Supplementary material for: Unveiling the global influence of tropical cyclones on extreme waves approaching coastal areas
Source: Nat Commun. 2024 Aug 3;15:6593. doi: 10.1038/s41467-024-50929-2 (PMC11297989; doi:10.1038/s41467-024-50929-2)
Supplement: Supplementary file 1 — Supplementary Information [file 41467_2024_50929_MOESM1_ESM.pdf]

Unveiling the Global Influence of Tropical Cyclones on Extreme Waves approaching Coastal Areas

Swen Jullien[1], Jérôme Aucan[2], Elodie Kestenare[3], Matthieu Lengaigne[4], Christophe Menkes[2]

1 Univ Brest, Ifremer, CNRS, IRD, LOPS, F-29280 Plouzané, France
2 ENTROPIE (IRD, CNRS, Ifremer, Université de la Réunion, Université de la Nouvelle-Calédonie), Nouméa, New Caledonia
3 Université de Toulouse, LEGOS (IRD/CNES/CNRS/UT3), Toulouse, France
4 MARBEC, University of Montpellier, CNRS, Ifremer, IRD, Sète, France
Corresponding author: Swen Jullien, swen.jullien@ifremer.fr

Supplementary Information

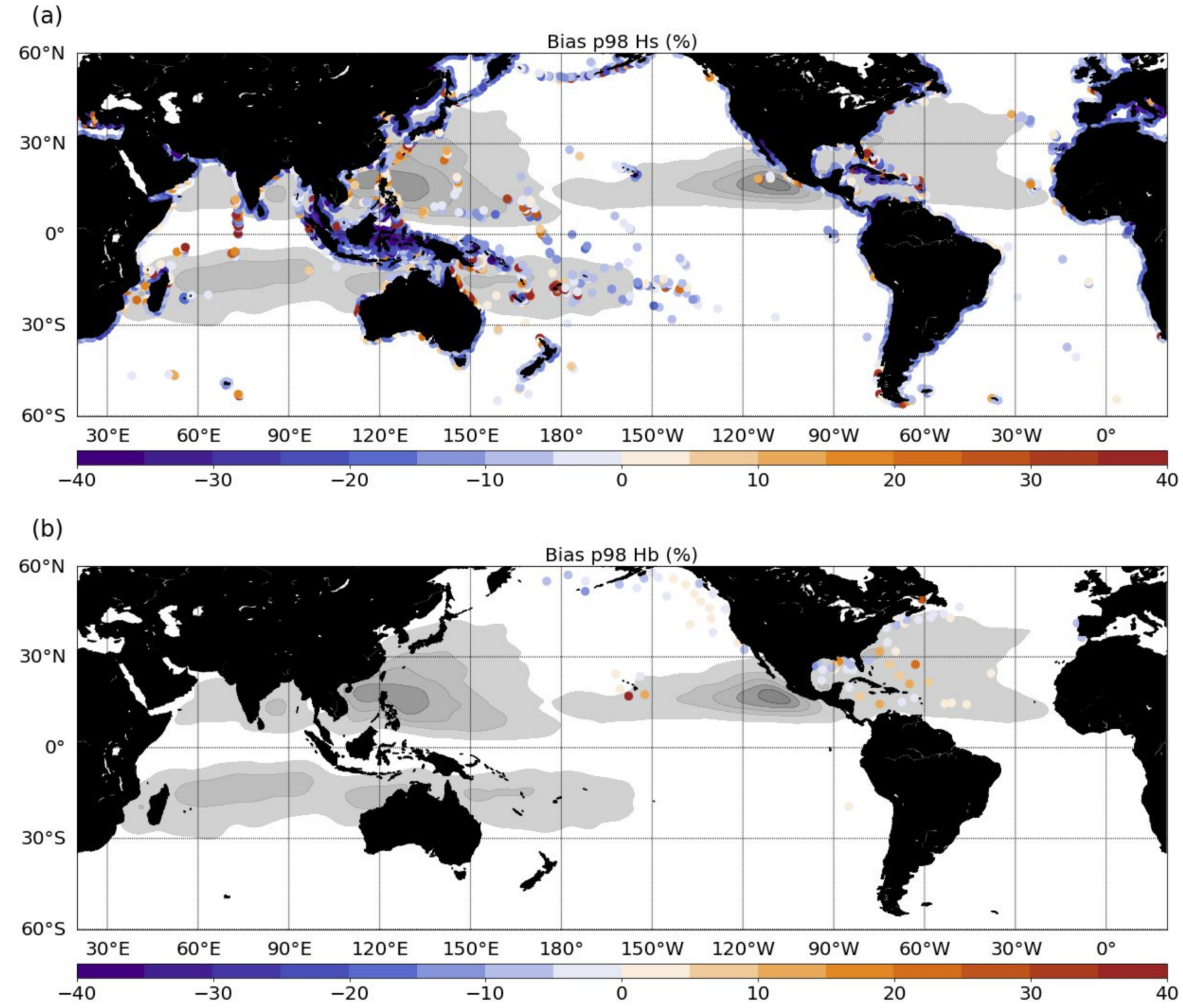

Fig. S1: Normalized bias (%) between the simulation including Tropical Cyclone (TC) forcing (CYCL) and observations. (a) Bias of the 98th percentile of significant wave height, Hs, compared with satellite observations over the 1992-2017 period, (b) bias of the 98th percentile of Hb compared with buoy observations over the 2012-2016 period. Cyclone density is indicated in gray shading.

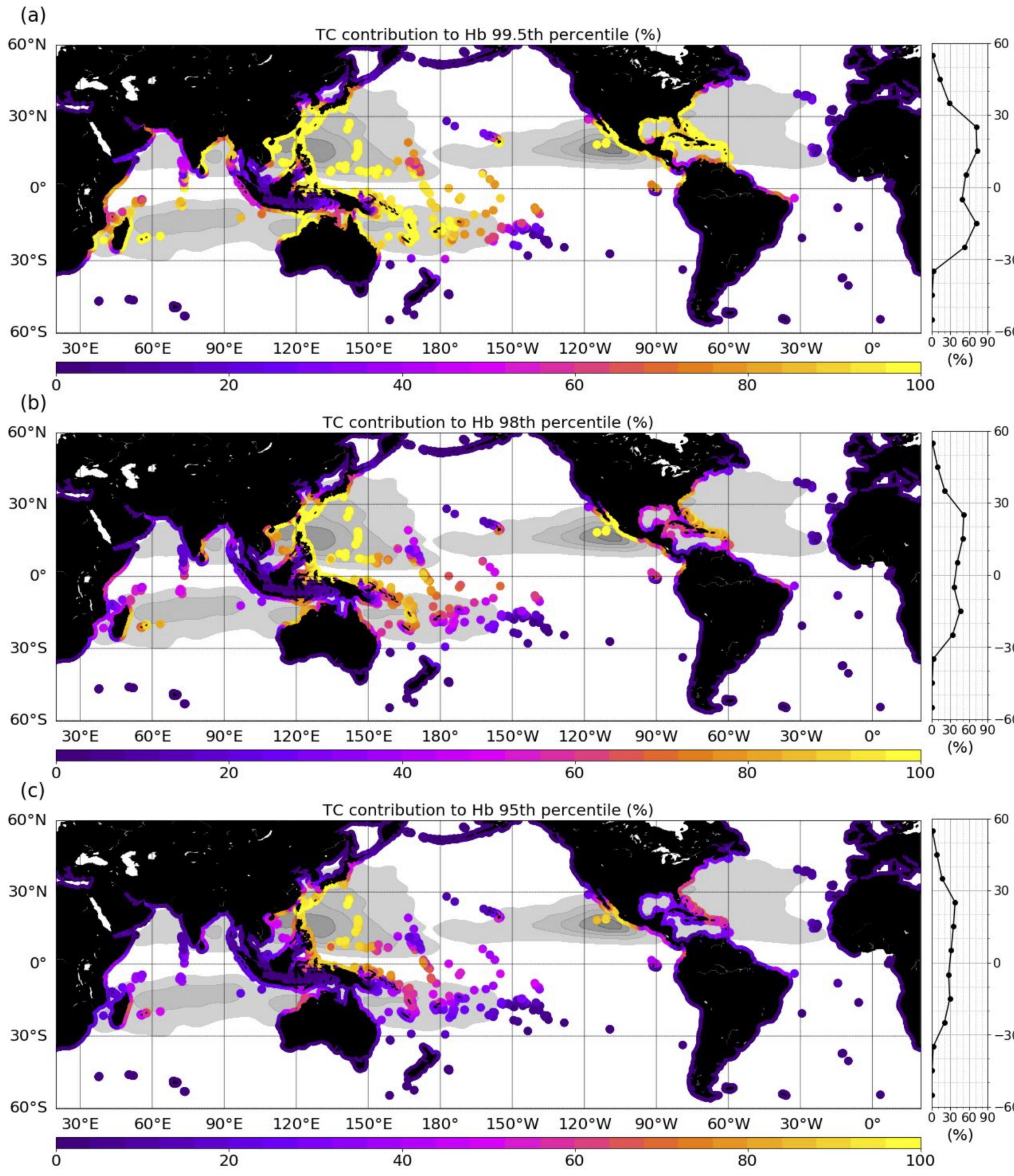

Fig. S2: Contribution of Tropical Cyclones (TCs) to extreme wave events. The contribution is computed as the relative difference (in %) in the number of occurrences of breaking wave heights (Hb) above a given threshold between the simulation including TC forcing (CYCL) and the simulation excluding TC forcing (NOCYCL). The threshold is fixed at (a) Hb 99.5^{th} percentile, (b) 98th percentile, (c) 95th percentile. Side plots on the right represent zonal averages of the corresponding map on the left. The cyclone density is indicated with gray shading.

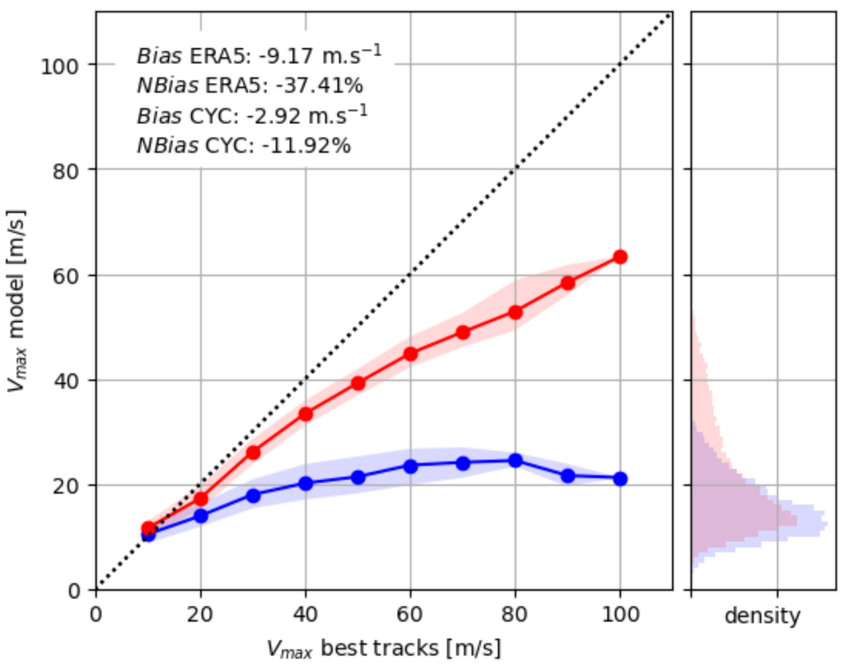

Fig. S3: Comparison of Best Tracks and model forcing maximum intensity in tropical cyclones. Red is for our blended wind forcing including parametric tropical cyclones (CYCL), blue is for ERA5 winds. Dots represent the binned median and shading the lower-upper quartile interval; the density of points is indicated in the side plot. TC maximum wind speed appears strongly underestimated in ERA5, in CYCL a slight underestimation arises while reaching the most intense TCs due to the interpolation of the wind field on the 1/2° resolution WAVEWATCH III model grid that smoothes the maximum wind.
